# 553. Pleural Space Infection Microbiology as Assessed Using a Clinical Targeted Metagenomic Sequencing-Based Assay on Pleural Fluid, and Proposal of the Term, “FuSion” ( *Fusobacterium nucleatum* group, *Streptococcus intermedius* and oral normal microbiota) to Describe the Most Common Bacteria Identified in Community-Acquired Pleural Space Infections

**DOI:** 10.1093/ofid/ofad500.622

**Published:** 2023-11-27

**Authors:** Judith Alvarez Otero, Jayawant Mandrekar, Matthew Wolf, Jordan Starkey, Eva Carmona, Ruben Dyrhovden, Øyvind Kommedal, Robin Patel

**Affiliations:** Mayo Clinic, Rochester, Minnesota; Mayo Clinic, Rochester, Minnesota; Mayo Clinic, Rochester, Minnesota; Mayo Clinic, Rochester, Minnesota; Mayo Clinic, Rochester, Minnesota; Haukeland University Hospital, Bergen, Norway, Bergen, Hordaland, Norway; Department of Microbiology, Haukeland University Hospital, Bergen, Norway, Bergen, Hordaland, Norway; Mayo Clinic, Rochester, Minnesota

## Abstract

**Background:**

Definition of the microbiology of pleural space infection has been challenging due to the poor yield of conventional culture, a challenge that has led to recommendations for broad spectrum antimicrobial therapy for these infections. Since August 2019, we have offered a 16S ribosomal RNA gene PCR assay followed by Sanger or next generation sequencing, or reported as negative, depending on the PCR signal strength (e.g., crossing point) in clinical practice at Mayo Clinic.

**Methods:**

Here, we describe the microbiologic and clinical findings of 57 pleural fluids testing positive by 16S rRNA gene PCR/sequencing from August 2020 to January 2023. Demographic data and clinical features, and results of pleural fluid and blood cultures were recorded. Analysis was performed using SAS software version 9.4 (SAS Inc).

**Results:**

Median patient age was 63 years (range 5-83 years) and 75% were men. Pleural space infection was community-acquired in 84%. The most frequent symptom was dyspnea (80%), followed by chest pain (52%). 89% of patients had received antibiotics in the two weeks preceding specimen collection. Radiological consolidation was observed in 63% and pleural fluid was loculated in 58%. Pleural fluid and blood cultures were positive in 32 and 11%, respectively. The microbiology found with the sequencing assay is shown in the table. 23/57 (40%) detections were polymicrobial. *Fusobacterium nucleatum* group and/or *Streptococcus intermedius* were detected in 31/57 (54%) cases [including 28/48 (58%) in the subset with community-acquired infection], with additional facultative and/or anaerobic species in various combinations also found in 17/31 (55%) of these cases.

**Figure:**
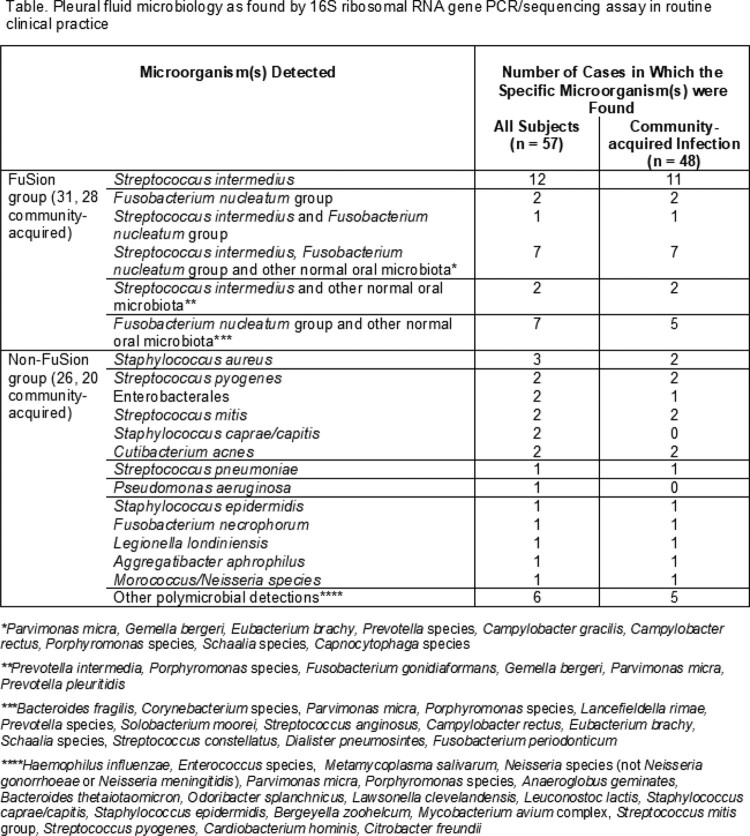

**Conclusion:**

Based on analysis of pleural fluid using a targeted metagenomic sequencing assay in clinical practice, the most frequent organism profile involved in community-acquired pleural space infection is a combination of *Fusobacterium nucleatum* group and/or *Streptococcus intermedius*, with or without other normal microbiota, a grouping for which we propose the term FuSion (*Fusobacterium nucleatum* group, *Streptococcus intermedius* and oral normal microbiota).

**Disclosures:**

**Eva Carmona, MD**, boehringer ingelheim: Advisor/Consultant **Øyvind Kommedal, n/a**, Pathogenomix inc.: Advisor/Consultant|Pathogenomix inc.: Stocks/Bonds **Robin Patel, MD**, Abbott Laboratories: Advisor/Consultant|Adaptive Phage Therapeutics: Grant/Research Support|Adaptive Phage Therapeutics: Mayo Clinic has a royalty-bearing know-how agreement and equity in Adaptive Phage Therapeutics.|BIOFIRE: Grant/Research Support|CARB-X: Advisor/Consultant|ContraFect: Grant/Research Support|Day Zero Diagnostics: Advisor/Consultant|HealthTrackRx: Advisor/Consultant|Mammoth Biosciences: Advisor/Consultant|Netflix: Advisor/Consultant|Oxford Nanopore Technologies: Advisor/Consultant|PhAST: Advisor/Consultant|See details: Patent on Bordetella pertussis/parapertussis PCR issued, a patent on a device/method for sonication with royalties paid by Samsung to Mayo Clinic|See details: continued, patent on an anti-biofilm substance issued|TenNor Therapeutics Limited: Grant/Research Support|Torus Biosystems: Advisor/Consultant|Trellis Bioscience, Inc.: Advisor/Consultant

